# Periosteal Distraction Osteogenesis: An Effective Method for Bone Regeneration

**DOI:** 10.1155/2016/2075317

**Published:** 2016-12-18

**Authors:** Danyang Zhao, Yu Wang, Dong Han

**Affiliations:** ^1^Department of Plastic and Reconstructive Surgery, Shanghai Ninth People's Hospital, Shanghai Jiao Tong University School of Medicine, No. 639, Zhizaoju Road, Shanghai 200011, China; ^2^Department of Geriatrics, Shanghai Ninth People's Hospital, Shanghai Jiao Tong University School of Medicine, No. 639, Zhizaoju Road, Shanghai 200011, China

## Abstract

The treatment of bone defects is challenging and controversial. As a new technology, periosteal distraction osteogenesis (PDO) uses the osteogenicity of periosteum, which creates an artificial space between the bone surface and periosteum to generate new bone by gradually expanding the periosteum with no need for corticotomy. Using the newly formed bone of PDO to treat bone defects is effective, which can not only avoid the occurrence of immune-related complications, but also solve the problem of insufficient donor. This review elucidates the availability of PDO in the aspects of mechanisms, devices, strategies, and measures. Moreover, we also focus on the future prospects of PDO and hope that PDO will be applied to the clinical treatment of bone defects in the future.

## 1. Introduction

Bone regeneration is a major challenge in the reconstructive surgery field. The commonly used therapies for bone defects are bone graft substitutes, guided bone regeneration (GBR), and distraction osteogenesis (DO). Autologous bone graft, the gold standard for the treatment of bone defects, although it can avoid the immune-related complications, is limited by donor, pain, morbidity, secondary trauma surgery, bone resorption, and osteonecrosis [1, 2]. Other graft substitutes, such as allogenic bone and biosynthetic materials, have the problem of biocompatibility, which often lead to infection, immune rejection, and implant displacement [3]. GBR is a technique that uses a layer of high molecular biological membrane as a barrier to cover bone defect; it can stop the entry of irrelevant tissues or cells and maintain the stability of blood clots to let the coagula fill the defect gap [4]. DO, also known as “the endogenous bone tissue engineering,” forms new bone by gradually separating two bone segments on the condition of osteotomy or corticotomy [5, 6]. This approach can generate sufficient osseous mass, but it is invasive for human body and has a long treatment cycle; it also easily causes bone nonunion and fibrous ossification. Schmidt et al. [7] were the first to confirm the histological formation of new bone by periosteal distraction without corticotomy, and the conception of periosteal distraction osteogenesis (PDO) gradually arose from it.

**Figure 1:**
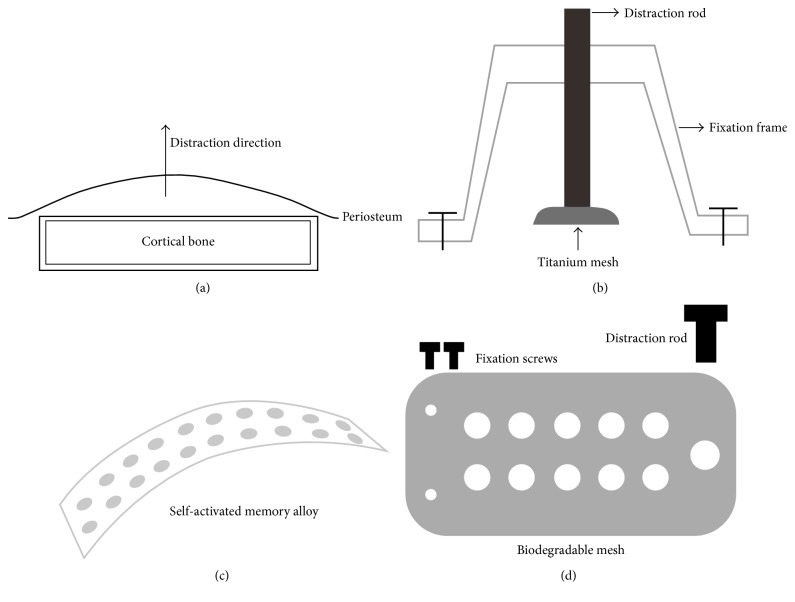
The mechanism (a) and devices (b, c, d) of PDO. (a) PDO creates an artificial space between the bone surface and periosteum to generate new bone by expanding the periosteum, muscle, and skin at the same time. (b) U-shaped distractor composes of three different parts: fixation frame, distraction rod, and titanium mesh. Bilateral fixation legs can be fixed rigidly to the surface of cortical bone by titanium screws, and then through the rotation of middle distraction rod, the titanium mesh can be lifted off the ground of bone and distract the periosteum simultaneously. (c) SMA leaves out distraction screws. (d) Biodegradable PLLA/HA mesh instead of titanium mesh for distracting periosteum.

PDO is a breakthrough after DO; it is the combination of tissue expansion and GBR, which creates an artificial space between bone surface and periosteum by expanding the periosteum, muscle, and skin at the same time (Figure 1(a)). It can avoid the occurrence of immune-related complications and solve the problem of insufficient donor; it also does not need corticotomy comparing with DO. A large number of researchers have explored the feasibility and superiority of PDO through many animal experiments (Table 1). This review will discuss the effectiveness of PDO in the aspects of mechanisms, devices, strategies, and measures.

## 2. Mechanisms of PDO

Periosteum plays a significant role both in DO and in PDO. The osteogenicity of periosteum has already been proved in DO. Kojimoto et al. [8] implanted an orthofix at tibiofibular junction in rabbit and found that removing periosteum could obviously affect callus formation, suggesting that periosteum is important for DO, even more important than corticotomy; another study also supported this finding [5]. Sun and Herring [9] regarded that the periosteal injury would inhibit the early period of mandibular DO site healing. Besides, Takeuchi et al. [10] proved that there was more newly formed bone in periosteum retention group compared with that in the periosteum excision group by micro-CT, and the preservation of periosteum could not only prevent the resorption of external bone, but also maintain vertical height of mandible during DO. Furthermore, Yin et al. [11] also stressed the necessity of maintaining the integrity of periosteum in the installation of dental implant distractor.

As we all know, the periosteum is composed of two different parts. The outer layer is also called fibrous layer, which is closely integrated by collagen fibers; it is rich in blood vessels and nerves and has nutritional and sensory function. The inner layer is also called cambium layer, which is arranged in order by osteocytes; it is involved in the growth and proliferation of bone and has the ability of osteogenicity [12]. The periosteum is rich in bone progenitor cells which can differentiate into osteoblasts in the process of periosteum stretch [13]. An early study [14] demonstrated that the mechanical strain can stimulate human periosteal cells to increase the expression of Runt-Related Transcription Factor 2 (RUNX2) and upregulate some osteogenic and angiogenic growth factors, such as transforming growth factor-*β* (TGF-*β*), basic fibroblast growth factor (b-FGF), vascular endothelial growth factor (VEGF), and platelet derived growth factor (PDGF). Thus it is theoretically possible to produce new bone only by periosteum distraction without corticotomy, namely, PDO. The slow and stable tension can activate the mesenchymal stem cells (MSCs) to differentiate into osteoblasts with high activity and even calcify to mature bone tissue.

There is another theory that supports the feasibility of PDO. Stevens et al. [16] took advantage of the osteogenicity of rabbit tibial periosteum to acquire new bone and successfully repaired the contralateral tibial defects. They called the artificial space between the periosteum and the tibia “in vivo bioreactor,” which creates a space in the body and uses the organism's own potency to regenerate tissue for repair [39]. The periosteum is equivalent to a physical barrier that effectively prevents other soft tissues from invading and is also conducive to the supplement of bone cells. Using this method to construct tissues is similar to bone autograft; it can be achieved by body's own healing mechanism and regenerative potency.

To sum up, the mechanism of PDO lies in the formation of an “in vivo reactor” between the periosteum and cortical bone. Using the osteogenicity of periosteum, it not only releases osteogenic cells and factors during the distraction, but also creates an independent space for bone regeneration.

## 3. The Designs and Materials of Distraction Devices

In order to obtain a good result of osteogenesis, we must carry out a stable and sustained stretch for periosteum. Researchers often used different designs and materials to analyze the effect of PDO (Table 1). With the progress of science and technology, the distraction devices are gradually evolving.

At first, Kostopoulos and Karring [15] implanted the Teflon (PTFE) capsules at mandibular ramus of rats; the capsules could avoid the interference of the surrounding soft tissue, whereas they block the contact between the periosteum and the cortex unfortunately. They suggested that the periosteal distraction devices should be perforated in order to maintain the communication between the periosteum and cortical bone.

Schmidt et al. [7] then used a U-shaped distractor (Figure 1(b)) to stretch the periosteum of rabbit mandible and acquired new bone height of average 2.86 ± 0.56 mm, and the U-shaped distractor has been improved later [18, 21, 26, 33, 36, 40]. The U-shaped device is usually made of titanium alloy or stainless steel with advantages of high strength and corrosion resistance. It often has three different parts; they are fixation frame, distraction rod, and titanium mesh. Bilateral fixation legs can be fixed rigidly to the surface of cortical bone by titanium screws. Through the middle distraction rod, the titanium mesh will be lifted off the ground of bone and distracts the periosteum simultaneously. The speed and frequency of U-shaped distractor can be controlled manually, but it often causes damage to the soft tissues, especially to the integrity of periosteum. Screw looseness and mesh disengagement also occasionally occur; thus further improvement is needed. Nowadays, the distraction devices are continuously modifying; many researchers only used a titanium mesh and few screws to achieve the same effect; those distraction devices not only simplified the operation process, but also reduced the damage to soft tissues [17, 19, 23–25, 28–30, 41].

To overcome the manual operation problem, Abrahamsson et al. [22, 42, 43] put a self-inflatable osmotic expander under the mandibular periosteum of rabbits and then placed a preformed scaffold that was filled with autogenous bone graft or bone substitute; finally the distraction device acquired newly formed bone after three months. Yamauchi et al. [31, 32, 38] then designed a new type of self-activated memory alloy (SMA) (Figure 1(c)); it does not need distraction screws and thus solves the complications with the minimal invasion. Nonetheless, the accuracy and controllability of the above two kinds of expansion devices were relatively poor. It is difficult to guarantee the accuracy of quantitative distraction without damage to the osteogenic potential of periosteum.

Besides the designs, the materials of distraction devices are changing rapidly. In one study, biocompatible gel was injected into the space between the periosteum and tibia to distract the tibial periosteum [16]. The gel was completely degraded after 2 weeks, and there was no obvious difference between the new bone and tibial cortex by the time of 8 weeks. Yamauchi et al. [20, 44] implanted a highly purified beta-tricalcium (*β*-TCP) block on the lateral surface of the beagle dog mandible. With the degradation of material, the *β*-TCP block was gradually replaced by new bone. In another experiment, the graft which was implanted in the distracted area between the alveolar bone and *β*-TCP block could stably exist [45]. Zakaria et al. [27] then tried to use biodegradable poly-L-lactide/hydroxyapatite (PLLA/HA) mesh (Figure 1(d)) to replace the titanium mesh for distracting periosteum. Recently, Dziewiecki et al. [37] compared nondegradable titanium to degradable devices (poly-DL-lactide and polyglycolic acid) in PDO; they also proved that degradable devices could produce new bone and there were no significant differences in the amount of newly formed bone between titanium and degradable materials.

Those above measures are similar to the in vivo bone tissue engineering, yet not requiring seed cells and exogenous growth factors. They solve the problem of second operation for pulling the device out, but the choice of biodegradable materials (biodegradability and toxicity) and the stability of degradable materials need to be studied.

## 4. Effect of Distraction Strategies on the Formation of New Bone

**Figure 2:**
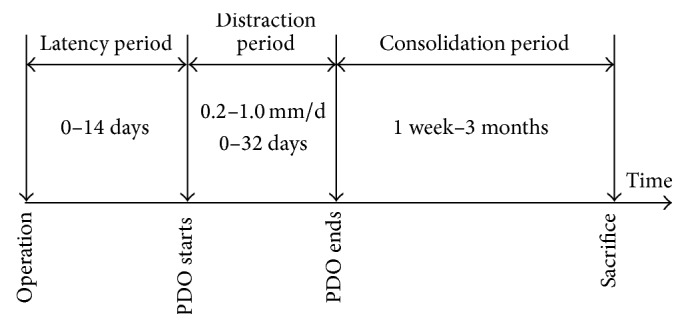
Protocol of PDO applied in different studies. The latency periods of PDO are different from 0 days to 14 days, the distraction periods of PDO are different from 0 days to 32 days, and the speed is 0.2–1.0 mm/d; the consolidation periods of PDO are different from 1 week to 3 months.

Similar to the traditional DO [46], the strategies of PDO can be divided into three stages: latency period, distraction period, and consolidation period (Figure 2). Different stages of PDO will affect the effect of osteogenesis, but the optimal parameters, including the distraction site, have not been obtained.

### 4.1. Distraction Sites

PDO was initially applied to the distraction of atrophic or edentulous mandible for increasing the height and width of alveolar ridge and was used for endosseous implant placement [47]. In the past, most of the distraction devices were placed at the internal and external sides of the mandible, but the alveolar gap was too narrow to perform the operation, and the devices would fall off because of animals' chewing action. Later, tibial periosteum was used to obtain new bone tissue [16]. Same as the mandibular bone, this method was limited by the size and space of osteogenesis, and the regenerated bone was insufficient to repair large bone defects. In order to solve these problems, Kessler et al. [17] implanted a titanium mesh and a screw on the forehead of pig and distracted the calvarial periosteum through the dynamic rotation of the screw. On the one hand, the skull bone was flatter than other bones and the periosteum of skull was thicker than other parts of the body; on the other hand, the area was adequate and easy to separate. Using the calvarial periosteum solves the problem of insufficient source of bone tissues.

The choice of sites determines the effect of distraction. Flat bone floor and tough periosteum will greatly improve the effect of PDO. Besides, the distraction site should be keep away from the incision place as far as possible to avoid the incremental tension in the process of distraction; otherwise, the wound will tend to have a dehiscence and result in the failure of the experiment.

### 4.2. The Length of Latency Period

The latency period refers to the intermission from the placement of device to the distraction. The traditional DO has a latency period of 5–7 days, while the latency periods of PDO are different from 0 days to 14 days (Figure 2) according to the difference of materials [46, 48]. In order to evaluate the effect of different latency periods on the PDO, 7-day latency period and 1-day latency period were compared [26], and the result showed that the average new bone masses were 2.62 cm^2^ and 3.26 cm^2^, respectively, but without significant difference, suggesting that bone tissue can be made by PDO using different latency periods. From another point of view, during the latency period, animals are gradually adapted to the device and the wound is also gradually healing, so it is recommended to wait for at least one week to proceed to the distraction.

### 4.3. The Speed and Frequency of Distraction

The distraction period is to separate the periosteum from the bone surface by a slow and persistent tension [46, 48]. According to Ilizarov's law of tension-stress, the speed of distraction for limb lengthening should be 1 mm every day [6]. Many researchers tend to take the speed of 0.2–1.0 mm/d in PDO (Figure 2). It is because that cells and nutrition supply simultaneously come from the two bone ends and the surrounding periosteum in the process of DO, while in PDO, these can only come from the basal bone and periosteum; thus the speed of 1 mm/d is relatively fast.

In a study [19] that used the speed of 0.25 mm/d and 0.5 mm/d to distract the periosteum, lower speed was found to be more favorable for new bone formation. However, Saulacic et al. [36] believed that the high speed of distraction might be beneficial to periosteal osteogenesis, although it was easy to cause the disruption of wound and exposure of the device. Zakaria et al. [27, 28] designed a new type of device, by means of the inclined structure; this device could be used to study the effect of different distraction rates at the same time. The result suggested that the optimal speed of distraction should be lower than 0.33 mm/d. Low distraction speed could reduce the invasion of the surrounding soft tissues; what is more, the newly formed bone would contain relatively thicker trabecular bone and less fat tissue.

As for the frequency of distraction, the frequency of once a day, four times a day, and sixty times a day were used to study the effect of different distraction speeds on limb elongation [6], and the result showed that 1 mm/d with four steps once a day was the best for DO. However, researchers often used the frequency of once a day or twice a day in the process of PDO, though there was no relevant literature to carry out a comparative study.

### 4.4. Dynamic Distraction versus Static Distraction

In the process of PDO, scholars carried out a lot of comparative works on the dynamic and static distraction. Static distraction achieves the desired height all at once, while dynamic distraction adopts a more gentle way to distract separately. Kessler et al. [17] found that the dynamic distraction was more favorable for early bone formation, and the newly formed bone was similar to the rows of micropillars in conventional DO, while the immediate distraction could just produce the woven bone. Lethaus et al. [24] put the distraction device under the calvarial bone, the result showed that the cumulative bone mass was about 66% in dynamic group and 67% in static group, and there were no significant differences between the two groups with regard to bone quality or quantity. Yamauchi et al. [38] used a SMA mesh device and an absorbable thread to conduct dynamic distraction; result showed that dynamic distraction group had higher volume of newly formed bone by comparing with simple SMA group.

Generally speaking, dynamic distraction might be more moderate, which can avoid damage to the osteogenic potential of periosteum when the stretch is excessive.

## 5. Measures to Promote the Formation of New Bone in PDO

Researchers conducted a lot of different explorations to increase the quality and quantity of osteogenesis, such as cortical bone perforation, MSCs administration, addition of different cytokines, and so on (Table 1). These technical improvements not only confirmed the feasibility of PDO, but also provided valuable information for improving PDO.

### 5.1. Cortical Bone Perforation

Cortical bone perforation is a big step in GBR, and PDO takes advantages of it to promote new bone formation. Exposure of cancellous bone by perforating on bone surface facilitates the release of MSCs from bone marrow or endosteum. Meanwhile, the increase of bleeding allows the angioblast cells to enter the space under periosteum, which is beneficial to the vascularization of newly formed bone.

Sencimen et al. [18] compared PDO with conventional DO in New Zealand male rabbits; they found that the newly formed bone was 14.4 mm^2^ in PDO group, compared with 25.4 mm^2^ in DO group; moreover, the formation of new bone in the DO group was more compact, while the formation of new bone in PDO group was rich in adipose tissue. Oda et al. [23] used a titanium mesh and a screw to distract the periosteum of mandible in rabbits; the average area of the new bone was 25.7 ± 5.1 mm^2^ and 12.9 ± 3.2 mm^2^ with or without decortication at 8 weeks after distraction period. The new bone could be seen under the whole mesh in the decortication group, but in the control group, the new bone could only be seen near the distraction screw; it might be because the local environment around the screw was similar to the experimental group, suggesting that cortical bone perforation was beneficial to bone expansion in PDO. Yamauchi et al. [32] united the technologies of bone perforation and SMA to carry on PDO to a height of 2.9 ± 0.5 mm and found that the new bone mass in experimental group was higher than that in the control group in each period. In the study of the osteogenetic effect in PDO, Saulacic et al. [30] considered that if the bone marrow cavity was not exposed, the new bone mainly depended on the periosteum; on the contrary, it would depend on bone cortex. We can conclude that cortical bone perforation influences the formation of new bone in PDO.

### 5.2. MSCs Administration

MSCs administration is actually the same as cortical bone perforation that can overcome the shortage of osteoblasts. MSCs not only participate in osteogenesis, but also produce enough VEGF to promote the formation of new blood vessels [49]. Sato et al. [25] injected MSCs into the space under the periosteum during PDO and the result showed that the volume, height, and degree of mineralization of the new bone in experiment group were higher than noninjected group, suggesting that MSCs administration could induce osteogenesis at periosteal distraction sites. However, it is necessary to explore the optimal injection time and frequency in the future.

### 5.3. Cytokines

In DO, the application of cytokines has obtained achievements, but in PDO, the study in this area is far from enough. VEGF, as a vascular growth factor, not only is conducive to the formation of blood vessels, but also can promote osteogenesis during the process of distraction [50, 51]; the injection of exogenous VEGF was proved to be beneficial to bone formation in PDO [21]. Another study [35] proved that the newly formed bone by PDO was more mature after adding platelet-rich fibrin (PRF). In addition, PDO could also induce the release of endogenous bone morphogenetic protein-2 (BMP-2) [36]. We have reason to believe that adding other biological factors which can promote the osteogenesis of DO, such as TGF-*β*, bFGF, and PDGF, can also improve the osteogenesis of PDO.

### 5.4. Other Measures

In recent years, researchers are still exploring other measures to improve PDO. One study found the bone formation was delayed and the new bone mineralization was insufficient in the ovaries-resected rabbits, but there was no significant histological difference compared with the control group [40], which indicated that the osteoporosis causing by decreased estrogen did not affect the new bone formation in PDO. Hyperbaric oxygen (HPO) was proved to be beneficial to PDO [33]. HPO therapy could improve the oxygen partial pressure in the blood and tissues, which could promote the synthesis of bone [52]. Kahraman et al. [34] made a local application of simvastatin when implanted distraction device, but there was not enough evidence to show that the use of lipid-lowering agents can promote the formation of new bone in PDO.

## 6. Future Directions and Prospects

The choice of materials, devices, and strategies is all variables in preclinical studies; for future preclinical work, those variables should be tuned to further optimize outcomes. The periosteum is a deeper implantation site and provides less available volume for osteogenesis, so the implantation site is very important; the periosteum should be thicker and enough implantation area is also needed. PDO still has a long period; it is necessary to promote the formation of new bone in PDO. MSCs and growth factors are promising; cortical bone perforation should be careful because it is hard to control and thus easily cause damage to the bone.

The biodegradable distraction devices seem to have advantages in PDO; nanomaterial is a potential candidate, because it can deliver drugs, growth factors, and genes with high efficiency [53, 54], which can be used for promoting cell proliferation, survival, and differentiation in bone regeneration. Future research should focus on the biodegradability, toxicity, and the stability of biodegradable materials. Besides, three-dimensional (3D) printing is a new technique with great potential in regeneration of tissues and organs [55, 56]; the distraction devices can be designed accurately by 3D printing technique to form complex shapes; what is more, 3D printing can design different sizes of holes in distraction devices to keep the communication between the periosteum and the cortex. On the whole, 3D printing technique designed biodegradable materials can combine with stem cells, growth factors, regenerative drugs, or other measures to produce sufficient amount of bone tissue; this might have great potential to achieve functional and aesthetic repair for bone defects.

## 7. Conclusion

There are still many disputes in the treatments of bone defects; as outlined above, PDO undoubtedly has fine application prospect. This review elucidates the advantages of PDO in the formation of new bone from the aspects of mechanisms, devices, strategies, and measures. At present, the technology of PDO has been used in the treatments of atrophic alveolar ridge and cleft palate, but there exist rare clinical reports; this might be attributed to device instability, soft tissues injury, infection, and other complications. Theoretically, newly formed bone by PDO can be applied to the bone defects in all parts of the body caused by hyperparathyroidism, calcium metabolism disorder, rickets, trauma, infection, and congenital malformation or other pathological conditions.

## Figures and Tables

**Table 1:** Summary of preclinical animal experiments.

Authors	Distractor	Animal	Position	Latency period	Distraction period	Consolidation period	Factor	Year	Reference number
Kostopoulos and Karring	PTFE capsule	Rat	Mandibular ramus	0 days	Immediate distraction	7, 14 days and 1, 2, 4 weeks	Different opening direction	1995	[15]

Schmidt et al.	Titanium mesh	Rabbit	Lateral surface of the mandible	7 days	7 mm over 15 days	Days 28, 35, 42, 56	None	2002	[7]

Stevens et al.	Calcium-alginate gel	Rabbit	Anteromedia aspect of metaphyseal and diaphyseal tibia	0 days	Immediate distraction	6, 8, 12 weeks	TGF-*β*/FGF-2	2005	[16]

Kessler et al.	Titanium mesh	Minipig	Forehead region	5 days	0.5 mm/d for 10 days	7, 17, 45 days	In comparison with IE	2007	[17]

Sencimen et al.	Stainless steel device	Rabbit	Lateral surface of the mandible	7 days	0.25 mm twice a day for 10 days	15, 30, 60 days	In comparison with DO	2007	[18]

Estrada et al.	Plate supported by a screw	Rabbit	Forehead	10 days	0.25 mm/d or 0.5 mm/d until 8 mm	10, 20, 30, 40, 50, 60 days after distraction	Different distraction rates	2007	[19]

Estrada et al.	Distraction rod with basel staple and titanium plate	Dog	Intraoral in the four quadrants	10 days	0.22 mm/d for 22 days	90 days	None	2007	[19]

Yamauchi et al.	Highly purified *β*-TCP block	Dog	Lateral surface of the mandible	8 days	0.5 mm/d for 8 days	8 weeks	None	2008	[20]

Casap et al.	U-shaped device Made of stainless steel and titanium	Rabbit	Mandible	2 weeks	1 mm/d for 7 days	60 days	VEGF	2008	[21]

Abrahamsson et al.	Osmotic self-inflatable expander	Rabbit	Lower border of the mandible	0 days	Depend on the inflation rate of the expander	2 weeks	None	2009	[22]

Oda et al.	Titanium mesh	Rabbit	Mandible	7 days	0.5 mm/d for 8 days	4 and 8 weeks	Cortical bone perforation	2009	[23]

Lethaus et al.	Laser-perforated titanium	Minipig	Forehead region	3 days	0.5 mm twice per day for periods 5, 10, 15 days, respectively	14, 28, 42 days	Versus static shielding	2010	[24]

Sato et al.	Mesh plate	Rabbit	Parietal bone	7 days	20 days	3 weeks	Bone marrow stem cell administration	2010	[25]

Altuğ et al.	U-shaped distractor built from stainless steel	Rabbit	Lateral aspect of the mandible	1 day or 7 days	0.25 mm twice a day for 10 days	15, 30, 60 days	Different latency periods	2011	[26]

Zakaria et al.	Biodegradable mesh (PLLA/HA)	Rabbit	Calvarial bone	7 days	0.5 mm every 12 hours for 5 days	4 and 6 weeks	Different distraction rates	2012	[27]

Zakaria et al.	Titanium mesh	Rabbit	Calvarial bone	7 days	0.5 mm every 12 hours for 5 days	4 and 6 weeks	Different distraction rates	2012	[28]

Saulacic et al.	Hemispherical disc	Rat	Calvarial bone	7 days	0.4 mm/d for 10 days	10, 20 days	None	2013	[29]

Saulacic et al.	Occlusive distraction plate or perforation of the distraction plate	Rat	Calvaria	7 days	0.2 mm/d for 10 days	7 days	Different distractors	2013	[30]

Yamauchi et al.	Ni-Ti SMA	Rabbit	Forehead	14 days	Depend on the elasticity of SMA	3 and 6 weeks	None	2013	[31, 32]

Suer et al.	Custom-design device	Rabbit	Lateral surface of the mandibular corpus	7 days	0.25 mm Twice a day for 6 days	4 and 8 weeks	HBO	2014	[33]

Kahraman et al.	A new periosteal distractor	Rabbit	Periosteumeum at the forehead		7 days	0.35 mm/d for 10 days, 45 days	Simvastatin	2015	[34]

Pripatnanont et al.	Modified Hyrax device	Rabbit	Ramus and body of mandible	3 days	0.5 mm twice a day for 7 days	4 and 8 weeks	PRF	2015	[35]

Saulacic et al.	Custom made distraction device	Rabbit	Calvaria	7 days	0.25 or 0.5 mm/d for 10 days	1 week, 2 weeks, 2 months	Different distraction rates	2016	[36]

Dziewiecki et al.	Nondegradable titanium device and degradable devices (poly-DL-lactide and polyglycolic acid)	Minipig	Calvarial bone	0 days	Immediate distraction	12, 28, 42 days	None	2016	[37]

Yamauchi et al.	SMA mesh device and absorbable thread	Rabbit	Under the periosteum at the forehead	0 days	Time-released dynamic distraction	4 and 8 weeks	None	2016	[38]

IE = Immediate Elevation; DO = distraction osteogenesis; SMA = self-activated memory alloy; HBO = hyperbaric oxygen; PRF = platelet-rich fibrin; PTFE = Polytetrafluoroethylene; TGF-*β* = transforming growth factor-*β*; FGF-2 = fibroblast growth factor-2.
